# Integration of small RNAs and transcriptome sequencing uncovers a complex regulatory network during vernalization and heading stages of orchardgrass (*Dactylis glomerata* L.)

**DOI:** 10.1186/s12864-018-5104-0

**Published:** 2018-10-03

**Authors:** Guangyan Feng, Lei Xu, Jianping Wang, Gang Nie, Bradley Shaun Bushman, Wengang Xie, Haidong Yan, Zhongfu Yang, Hao Guan, Linkai Huang, Xinquan Zhang

**Affiliations:** 1 College of Animal Science and Technology, Sichuan Agricultural University, Chengdu, 611130 Sichuan Province China; 2 Agronomy Department, University of Florida, Gainesville, FL 32611 USA; 3 USDA-ARS Forage and Range Research Lab, Logan, UT 80751 USA; 4 College of Pastoral Agriculture Science and Technology, Lanzhou University, Lanzhou, 730020 Gansu Province China; 5 Department of Horticulture, Virginia Tech, Blacksburg, VA 24061 USA

**Keywords:** *Dactylis glomerata*, Flowering, miRNA, Orchardgrass, Transcriptome, Vernalization-response

## Abstract

**Background:**

Flowering is a critical reproductive process in higher plants. Timing of optimal flowering depends upon the coordination among seasonal environmental cues. For cool season grasses, such as *Dactylis glomerata*, vernalization induced by low temperature provides competence to initiate flowering after prolonged cold. We combined analyses of the transcriptome and microRNAs (miRNAs) to generate a comprehensive resource for regulatory miRNAs and their target circuits during vernalization and heading stages.

**Results:**

A total of 3,846 differentially expressed genes (DEGs) and 69 differentially expressed miRNAs were identified across five flowering stages. The expression of miR395, miR530, miR167, miR396, miR528, novel_42, novel_72, novel_107, and novel_123 demonstrated significant variations during vernalization. These miRNA targeted genes were involved in phytohormones, transmembrane transport, and plant morphogenesis in response to vernalization. The expression patterns of DEGs related to plant hormones, stress responses, energy metabolism, and signal transduction changed significantly in the transition from vegetative to reproductive phases.

**Conclusions:**

Five hub genes, c136110_g1 (*BRI1*), c131375_g1 (*BZR1*), c133350_g1 (*VRN1*), c139830_g1 (*VIN3*), and c125792_g2 (*FT*), might play central roles in vernalization response. Our comprehensive analyses have provided a useful platform for investigating consecutive transcriptional and post-transcriptional regulation of critical phases in *D. glomerata* and provided insights into the genetic engineering of flowering-control in cereal crops.

**Electronic supplementary material:**

The online version of this article (10.1186/s12864-018-5104-0) contains supplementary material, which is available to authorized users.

## Background

Flowering is a crucial physiological event for plant reproduction. The appropriate flowering time is essential for the avoidance of adverse natural conditions allowing for a successful completion of reproduction. Typically, cold-induced vernalization and long day periods following vernalization are the two major factors driving the transition from vegetative to reproductive development. Vernalization provides competence to flower after prolonged cold exposure [1]. In *Arabidopsis thaliana*, several genes conduct a regulatory loop for the initiation of flowering. Prior to vernalization, *FRIGIDA* (*FRI*) up-regulates the expression of *FLOWERING LOCUS C* (*FLC*), which represses the flowering promoter *FLOWERING LOCUS T* (*FT*) and lead to flowering delay [2]. When plants are exposed to cold conditions, the expression of *FLC* is inhibited by the high expression of *VERNALIZATION 1* (*VRN1*), *VERNALIZATION 2*(*VRN2*), and *VERNALIZATION INSENSITIVE 3*(*VIN3*) [3], thus flowering is stimulated. For winter cereals, vernalization induced by low temperatures is the major driving factor for developmental phase transition and flowering initiation. Lacking a clear genetic background, information from *A. thaliana* has been used to infer possible genes and gene interactions involved in flowering pathways in cereals. Subsequent studies identified *VRN1*, *VRN2*, *VIN3*, and *VRN3* (an *FT* ortholog) in wheat and barley [4]. The functions of flowering time orthologues have likely been modified due to gene duplication in the complex genome of cereal crops, and the mode of vernalization response may be discrepant [5, 6]. In cereals, vernalization response is confined by the expression level of *VRN2* [7]. Before vernalization, highly expressed *VRN2* represses the expression of *VRN3* and causes delayed flowering. Vernalization accelerates flowering via suppressing expression of *VRN2* by the accumulation of *VRN1* product, and thus *FT* expression is stimulated. However, while these studies have provided rich information on the control of flowering time, they have primarily focused on annual crop species. In perennial grasses, the genetic mechanism of flowering is still largely unknown.

It has been demonstrated that miRNAs and their targets play important regulatory roles in plant development such as flowering stage and adaptation [8, 9]. For example, miRNA156 and miRNA172 antagonistically regulate phase transitions by negatively regulating their target *SQUAMOSA*­promoter binding protein­like (*SPL*s) and *APETALA2-LIKE* (*AP2*), respectively [10]. AP2-like transcription factor levels are reduced when miRNA172 expression is induced by *SPLs* from the vegetative to the reproductive phase. In another study, miRNA157 was predicted to cleave SPLs to facilitate vegetative-reproductive transition [11]. Past research has suggested that several miRNAs modulate plant development by targeting *AUXIN RESPONSE FACTORS* (*ARF*s) such as miRNA160 and miRNA167 [12, 13]. Similarly, blocking miRNA396 dramatically affects inflorescence development by inducing the GRF6 and subsequently activating the downstream biological clades including AUXIN (IAA) biosynthesis and *ARFs* [14, 15]. In addition, the expression of miRNA159, 164, 319, and 444 were also found to be altered in a manner correlated with plant development and flowering [16–20]. However, previous studies of miRNAs regulating flowering mostly emphasized potential interactions of miRNA-targets. The existing results were insufficient to elaborate the functional shift and dynamic functional variations of miRNAs throughout the reproductive cycle.

Temperate grasses are important source for human food and animal feed [21]. Orchardgrass (*Dactylis glomerata*) is one of the most economically important forage cereals due to its high biomass, good shade tolerance, and high nutritional value [22]. Vernalization is required for orchardgrass to flower [23]. However, few attempts have been made to review sequential miRNA expression profiles in orchardgrass among critical developmental stages in vernalization. In addition, the whole transcriptome reflecting the vernalization, booting, and heading process have not been previously described. The ever-accelerating improvement of sequencing technology has made it possible systematically conduct analyses of both mRNAs and miRNAs in non-model plants [24]. Thus, deep sequencing of miRNAs and mRNAs was performed to investigate the continuous molecular dynamics and identify potential regulatory miRNA targeted circuit responses to vernalization and flowering. Furthermore, a coexpression network was constructed to detect the key regulators during vernalization. This research will facilitate the understanding of vernalization and flowering mechanisms of *D. glomerata* and provide valuable information regarding flowering control in perennial grasses. In addition, this research may supply more foundation information for the grass family (*Poaceae*) including economically important cereals.

## Results

### Transcriptome and small RNA sequencing in five developmental stages

A total of 905.9 million raw reads were generated from 15 libraries. After trimming, 865.8 million clean reads were retrieved, accounting for 129.87 Gb of sequencing data (Additional file 1: Table S1 and Table S2). More than 193.7 million raw reads were obtained from 15 small RNA libraries, and the raw reads ranged between 10.6 million and 17.8 million per library (Additional file 1: Table S3). After the removal of low-quality sequences, 188.3 million clean reads remained (Additional file 1: Table S4). In each sample, read lengths ranging from 18 to 30 bp were mapped to *D. glomerata* transcriptome data, and the average percentage of total clean reads mapped was 65.33% (Additional file 1: Table S5). The lengths of *D. glomerata* miRNAs ranged from 18 to 30 nt, and 24 nt miRNAs were the most frequent size class among the 15 libraries (Additional file 1: Table S6). The unique sequences were mapped to species precursors in miRBase20.0 (http://microrna.sanger.ac.uk/) by BLAST to identify known miRNAs. A total of 109 known miRNAs were identified in the 15 libraries, and 21 nt miRNAs accounted for 75.23% of the known unique miRNAs (Additional file 1: Table S7). In addition, 163 novel miRNAs were detected with lengths ranging from 18 to 25 nt. The most commonly found novel miRNA length was 21 nt (Additional file 1: Table S7). The frequency of the first base of mature miRNAs showed that the known miRNAs of 24 and 25 nt preferentially started with ‘A’ (96% and 54.41%, respectively). Other miRNAs preferentially started with ‘U’ (Additional file 2: Figure S1A). Among novel miRNAs, 24 and 25 bp novel miRNAs preferentially started with ‘A’ (70.98% and 60.56%, respectively), the 29 bp novel miRNAs preferentially started with ‘C’ (55.56%), and other novel miRNAs preferentially started with ‘U’ (Additional file 2: Figure S1B).

### Differentially expressed miRNAs and correlation analysis of expression profiles of miRNA-targets

**Fig. 1:**
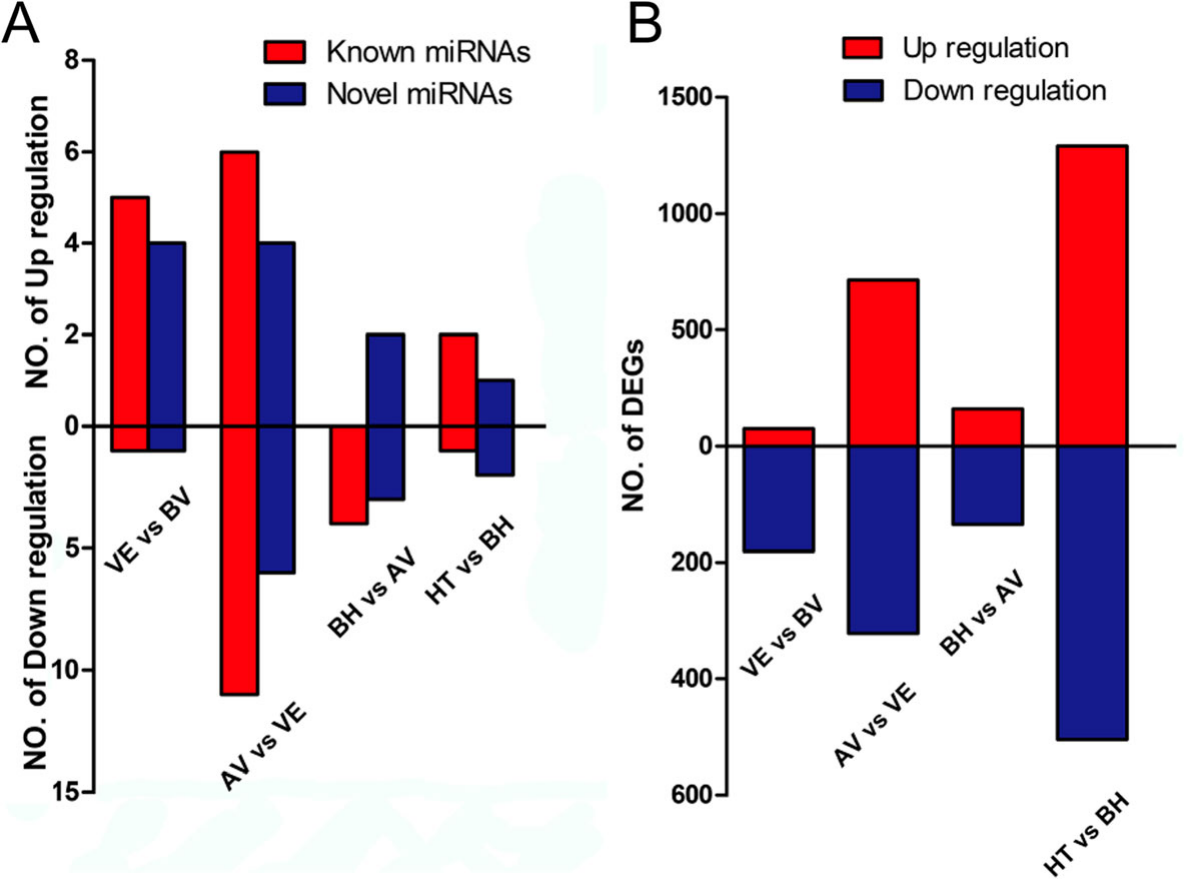
Summary of differentially expressed miRNAs and genes. **a** Differential expressed miRNAs in five comparisons. **b** Differential expressed genes in five comparisons. The abscissa represents the different stages, ordinate represents the gene number

To investigate the expression dynamics of the major miRNAs associated with the transition from vegetative period to reproductive period, differentially expressed miRNAs were detected in the following five comparisons: VE-BV (Vernalization-Before Vernalization), AV-VE (After Vernalization-Vernalization), BH-AV (Before Heading-After Vernalization), and HT-BH (Heading-Before Heading). In total, 53 miRNAs showed differential expression patterns among the five comparisons including 30 known miRNAs and 23 novel miRNAs. Of the 53 miRNAs, 11 were identified in the VE-BV comparison, 27 in AV-VE, nine in BH-AV, and six in HT-BH. Among these 53 differentially expressed miRNAs, 24 miRNAs were up-regulated while 29 miRNAs were down-regulated (Fig. 1a, Additional file 1: Table S8). The hierarchical clustering analysis showed that most miRNAs were preferentially expressed in a certain stage such as miR528, miR408, miR156, miR166, and miR1432 at the BV-stage; miR395, novel_72, novel_42, and novel_107 at VE stage; and novel_105, miR5072, and miR172 at the HT stage. In addition, the expression of several miRNAs significantly varied among different stages; for instance, miR166, novel_173, miR444, miR168, novel_16, miR398, miR397, and miR397 at the BV and VE stages; and miR1601, miR399, and miR394 at the BV and HT stages.

**Fig. 2:**
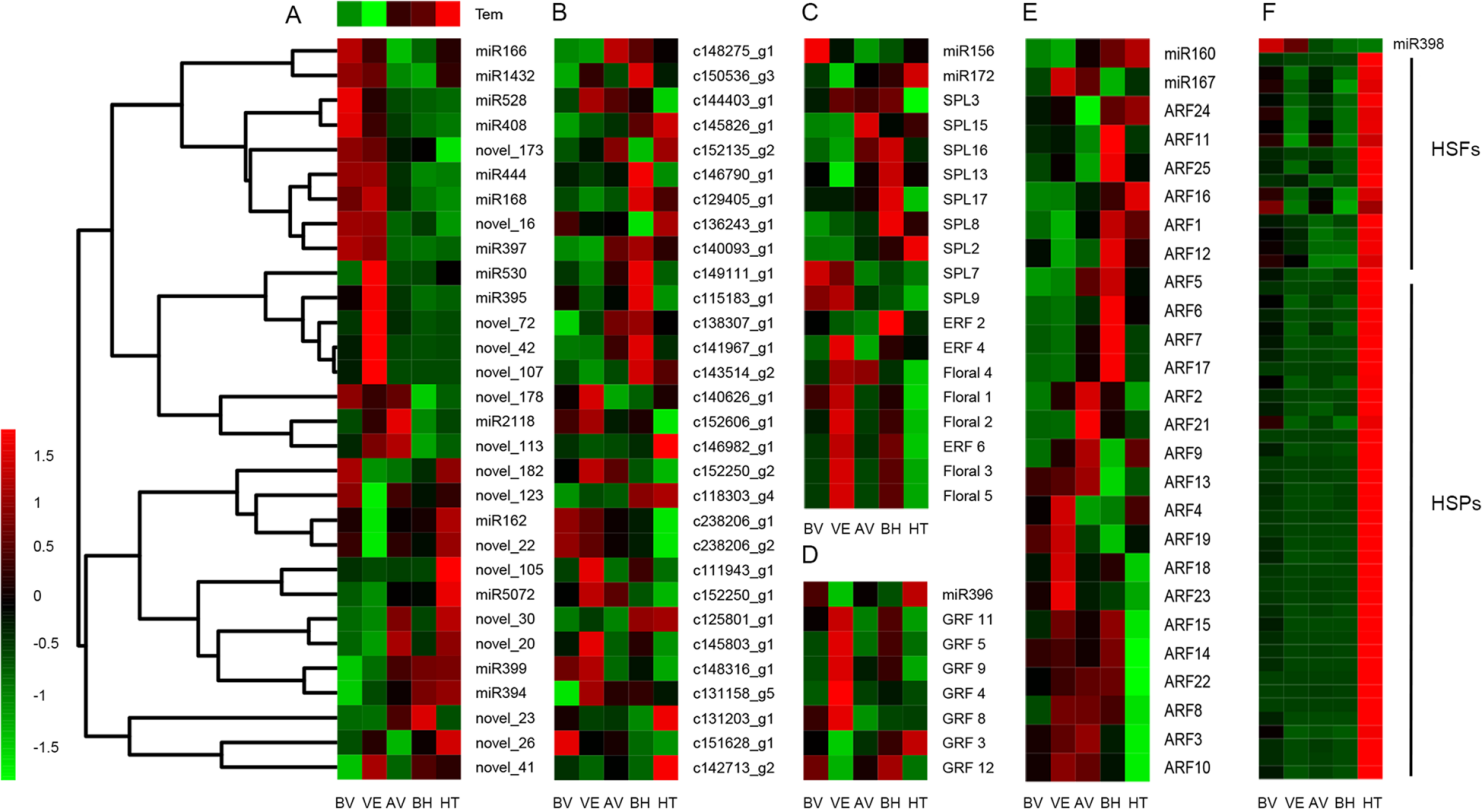
A combined view of expressions levels between differentially expressed miRNAs and their target genes in *Dactylis glomerata* L. at five developmental stages. **a** The differential expression of miRNAs and (**b**) their targets. **c** The expression profile of miR156, miR172, SPL family and AP2 family genes. **d** The expression profile of miR396 and GRF family genes. **e** The expression profile of miR160, miR167 and ARF family genes. **f** The expression profile of miR398, HSFs and HSPs family genes. The horizontal axis showed the different developmental stages and the vertical axis showed the miRNAs and genes. The separate heatmap labeled by “Tem” indicated the change of temperature

**Fig. 3:**
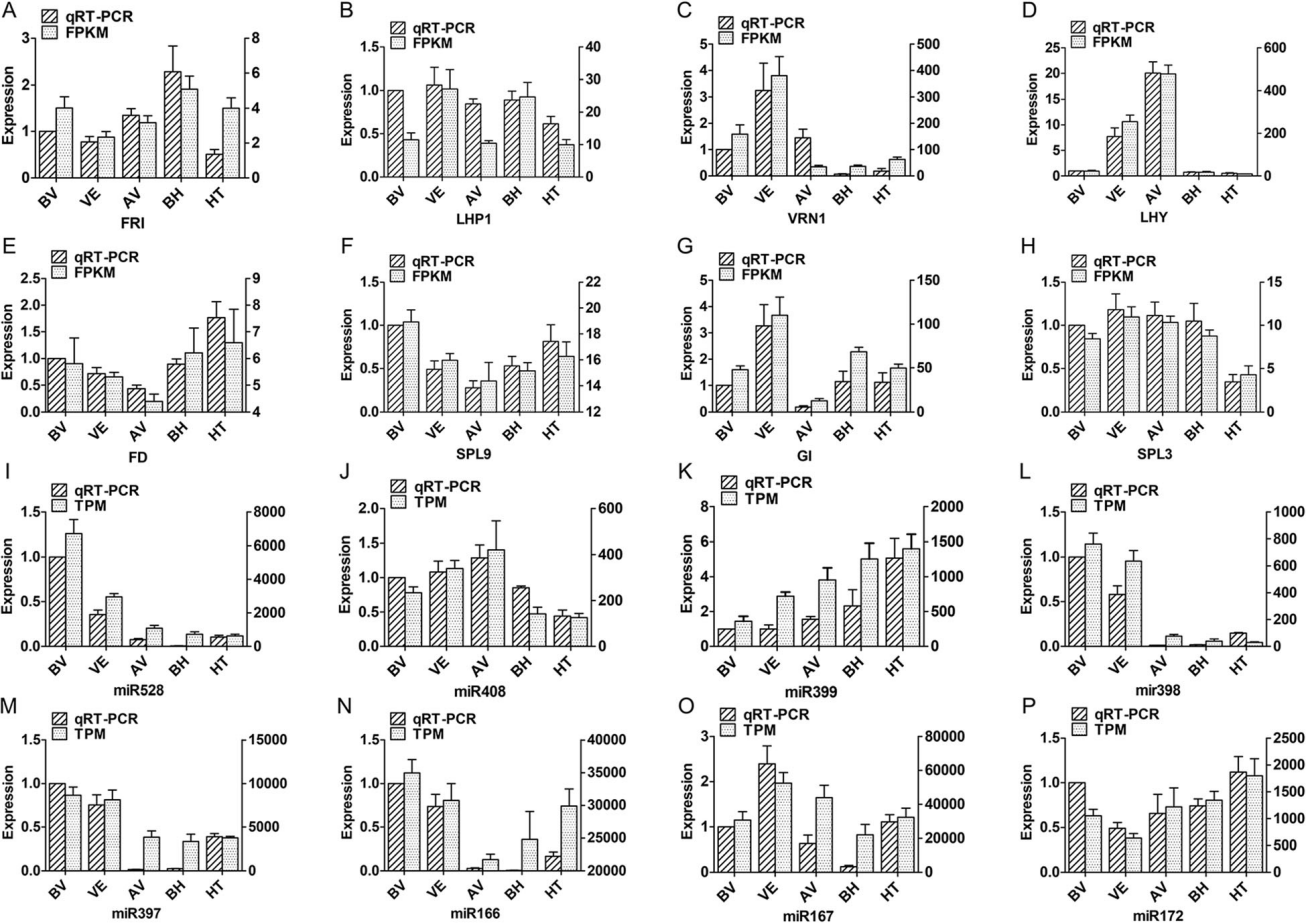
Verification by qRT-PCR of DEGs and differential expressed miRNAs. GAPDH and U6 were used as reference genes. The expression levels of each mRNA and miRNA were normalized by comparison with their expression with BV stage. The abscissa represents the different stages, ordinate represents the relative expression. The bar with oblique stripes represents the relative expression base on qRT-PCR results and the bar with faillette base on sequencing results. Figure **a**-**h** indicated the expression of eight DEGs and fig. **i**-**p** indicated 8 differential expressed miRNAs, the bottom title represents the gene and miRNA name, respectively

An expression analysis of miRNAs in combination with mRNA was performed to investigate the potential miRNA-target pairs with a significant influence on vernalization and floral development. A total of 71 DEGs were targeted by 39 differentially expressed miRNAs with a negative correlation in expression level [25] (Fig. 2a and b, Additional file 1: Table S9), of which several interesting miRNA-target pairs (miR156/172-SPLs/AP2s, miR396-GRFs, miR160/167-ARFs, and miR398-HSFs/HSPs) indicated that a transcriptional repression may be mediated by their corresponding miRNAs (Fig. 2c, d, e, and f).In addition, the qRT-PCR validation indicated the differentially expressed miRNAs and DEGs showed similar expression pattern to the sequencing data (Fig. 3).

### Function annotation and enrichment analysis of targets for miRNAs

The targets of specific miRNAs at the BV, VE, and AV stages were predicted to capture the functional properties related to vernalization, and 345 transcripts were anticipated to be the targets of 27 miRNAs (Additional file 3). The functional annotations indicated that several transcription factors were involved in growth regulation, such as *TCP2*, *PCF6*, *GAMYB*, *SPL16/17*, *MADS47/57*, *AIL1*, and *LHY*. Targets related to auxin efflux carrier components and calcium-transporters were also identified, which influence plant growth and flowering time by modulating the directional distribution of the phytohormone auxin and Ca^2+^ storage capacity within chloroplasts [26, 27]. The gene ontology (GO) analysis indicated that targets were significantly enriched (*P* < 0.05) in 122 GO terms including 68 biological process categories, 24 cellular component categories, and 30 molecular function categories (Additional file 3). In biological processes, the most frequent category was “transmembrane transport.” The results indicated that there were a series of transporters, sugar phosphate/phosphate translocator, metal-nicotianamine transporter, high-affinity nitrate transporter, and high-affinity iron ion transmembrane transport, which play essential roles in allowing plant response to intricate environmental signals [27, 28]. In addition, GO terms related to plant morphogenesis and functional differentiation, photosynthesis, immune system processes, and carbohydrate metabolism were also involved. This suggested that the miRNA targets related to transmembrane transport, carbohydrate metabolism, photosynthesis, immune system processes, and plant morphogenesis respond to vernalization and floral development.

### DEGs of transcriptome sequencing

**Fig. 4:**
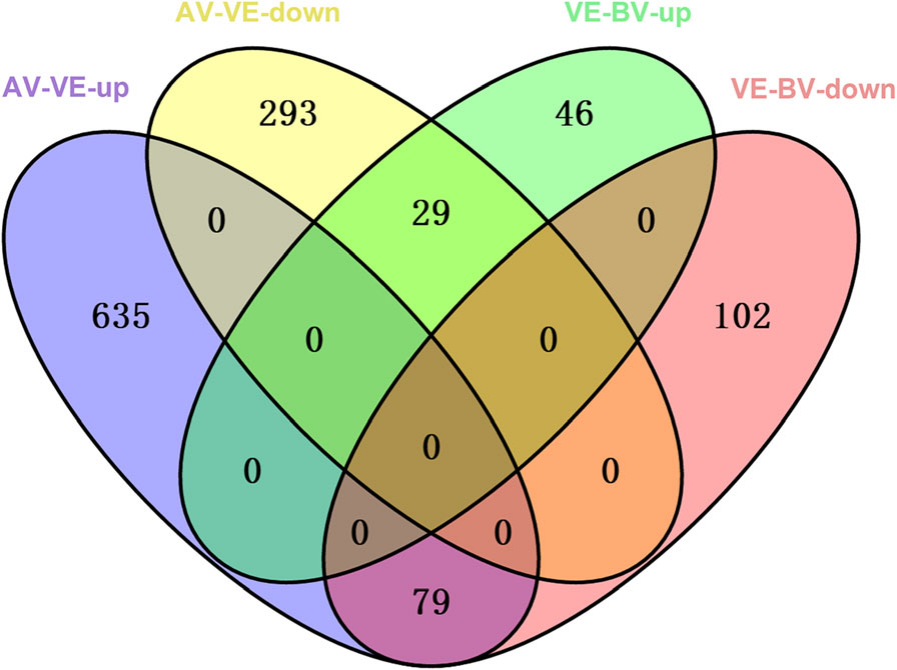
Relationships of changes in gene expression based on transcriptome sequencing. AV-VE-up, up-regulated genes at after vernalization stage compared with vernalization stage. AV-VE-down, down-regulated genes at after vernalization stage compared with vernalization stage. VE-BV-up, up-regulated genes at vernalization stage compared with before vernalization stage. VE-BV-down, down-regulated genes at vernalization stage compared with before vernalization stage

A total of 3,384 DEGs were detected including 256 DEGs in VE-BV, 1,036 DEGs in AV-VE, 295 DEGs in BH-AV, and 1,796 in HT-BH (Fig. 1b and Additional file 1: Table S10). Interestingly, 29 DEGs (including two ice recrystallization inhibition protein 4, an FT-like protein, and an ethylene-responsive element binding protein 2) were found up-regulated during vernalization and repressed afterwards (DEGs detected in VE-BV-UP and AV-VE-DOWN group). Seventy-nine DEGs (including a negative regulator of gibberellin signal DELLA protein *GAI*, three *bHLH* transcription factors, three heat stress transcription factors, and two heat shock proteins) were detected in both VE-BV-DOWN and AV-VE-UP (Fig. 4, Additional file 3).

Low temperature is the primary external factor of vernalization induction in temperate grasses. The expression of genes in response to external stimulation was up-regulated in VE-BV (such as ice recrystallization inhibition protein 4, light-inducible protein *CPRF2*-like, those related to plant hormones, such as cytokinin oxidase/dehydrogenase 2, ethylene-responsive transcription factor 73 (*ERF073*), *ERF3*, ethylene-responsive element binding protein 2 and *AUXIN*-related genes, and two flowering-related genes *FT-*like and flowering-promoting factor 1-like). The expression of 181 DEGs significantly declined (*P* < 0.01) from the BV stage to the VE stage including 25 DEGs for heat response such as *HSP20* family proteins and heat shock transcription factors (Additional file 3). In the VE stage, the expression of two *HSP20* family proteins, c125612_g1 and c112012_g1, decreased significantly (*P* < 0.01) with log_2_FC values relative to the BV stage of − 5.9908 and − 5.5984, respectively (Additional file 3). In VE-BV, the expression of seven plant hormone-related DEGs changed significantly (*P* < 0.01). The functions of two gibberellin-related genes with altered expression included gibberellin 2-beta-dioxygenase 8 and DELLA protein *GAI*. Ethylene-related genes with altered expression included *ERF024*, *ERF025*, *ERF027*, and *ERF1* (Additional file 3). A cytokinin-related gene, cytokinin riboside 5′-monophosphate phosphoribohydrolase, also decreased. In addition, several TFs were detected including *WRKY18*, *bHLH92*, *bHLH35*, GATA transcription factor 5, and NAC domain-containing protein 74 (*NAC74*). Remarkably, the expression of a flowering-delayed gene, *CONSTANS-LIKE 9* (c144305_g1), decreased significantly (*P* < 0.01) (Additional file 3).

**Fig. 5:**
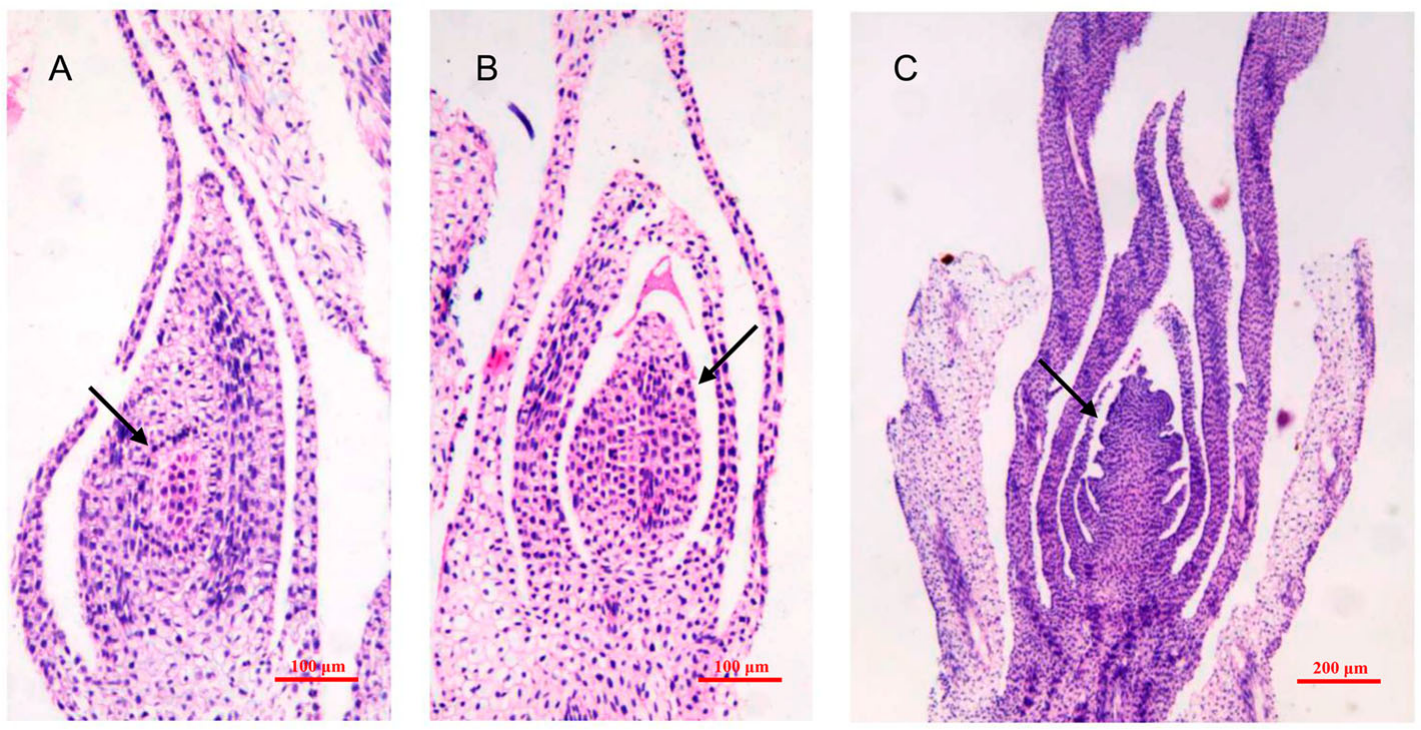
The floral primordium and young inflorescence of *Dactylis glomerata* after vernalization stimulation. **a** and (**b**) The floral primordium. **c** The young inflorescence. Arrows in (**a**) and (**b**) point to the floral primordium; arrows in (**c**) point to the young inflorescence

The temperature increased from stage VE to stage AV after a long period of chilling, causing a rapid acceleration of the growth of orchardgrass (Fig. 5). Our results showed 10 cellulose synthesis-related DEGs were up-regulated in the AV stage compared to the VE stage, most of which were annotated as cellulose synthase A. Meanwhile, the expression of seven DEGs with annotation of germin-like protein significantly (*P* < 0.01) increased in AV-VE (Additional file 3). It was also noted that the expression of *NAC* members elevated from the VE to AV stage (Additional file 3). Furthermore, nine *AUXIN*-related genes were detected among the up-regulated DEGs including IAA-amino acid hydrolase ILR1-like 2, auxin-responsive protein *IAA31*, indole-3-acetic acid-induced protein *ARG7*, indole-3-pyruvate monooxygenase *YUCCA11*, and indole-3-glycerol phosphate lyase. In contrast with *AUXIN*-related genes, the gibberellin-related genes gibberellin 3-beta-dioxygenase 2 and gibberellin 3-beta-dioxygenase 8 showed significant suppression in the AV stage (Additional file 3). Analogously, the expression of flowering-related genes *CONSTANS-LIKE 10*, *HEADING DATE 3A/3B*, and flowering-promoting factor 1-like protein 5 were also suppressed in the AV stage. The above results indicated that genes involved in stimuli-response; the plant hormones gibberellin, ethylene, and cytokinin; and flowering-related genes *FT*-like and *FPF1* were up-regulated in VE-BV, and genes-related to plant growth and *AUXIN* significantly varied between the AV-VE stages.

### Gene coexpression network analysis

**Fig. 6:**
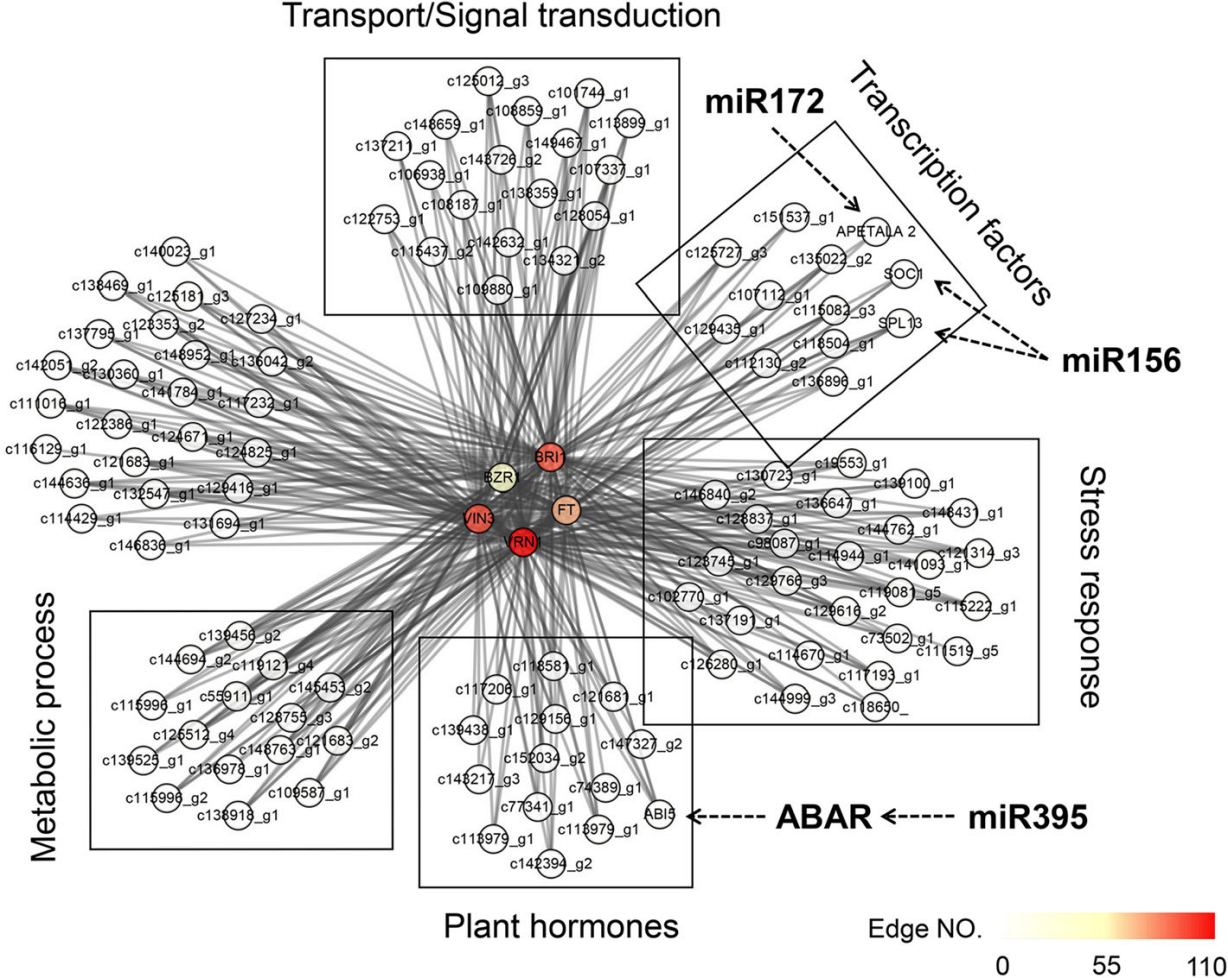
The coexpression subnetwork of BRI1, BZR1, VRN1, VIN3 and FT. The rectangular frame indicated the different functional categories. The dotted arrow showed the potential miRNA-target regulation

After excluding DEGs with low expression, 3,301 DEGs were used to construct a coexpression network to determine the genes with common expression trends across different samples in this study. The resulting network was composed of 12 distinct modules (labeled by different colors) (Additional file 4: Figure S2A). The size of the constructed genes for the 12 modules ranged from 1,506 (turquoise module) to 9 genes (grey module). The module-trait relationships analysis indicated that the pink module, black module, and brown module were highly correlated with the VE stage (Additional file 4: Figure S2B). To investigate the gene connection during vernalization, a subnetwork was identified with five hub genes in the brown module (Fig. 6, Additional file 3), in which two genes were related to brassinosteroid (BR) signal (*BZR1*and *BRI1*) and three genes were flowering regulators (*VRN1*, *VIN3*-like, and *FT*-like) relevant to the integration of veranlization and flowering pathways. The hub gene with the highest edge number (104 edges) was *VRN1*, a crucial regulator in vernalization response. In this subnetwork, 114 genes were involved in categories of transport process, transcription factors, stress response, protein modification, plant hormones, oxidation-reduction process, metabolic process, and carbohydrate metabolic process. Of the 114 genes, 14 genes were in the transport process category. Several potassium channel-related genes *KOR1* and *AKT1* were detected, which may function in perceiving and transferring environmental signals. In the stress response category, several genes related to environmental stimuli clustered together such as ice recrystallization inhibition protein 4, dehydrin, and heat stress transcription factor. In addition, genes in the plant hormone category, including 16 genes related to ABA, IAA, GA, ethylene, cytokinin, auxin, and brassinolide, may indicate the critical roles of plant hormones during vernalization. Furthermore, two floral homeotic proteins, *APETALA 2,* and a MADS-box protein *SOC1*-like, were also identified in this subnetwork.

## Discussion

### Differentially expressed miRNAs in developmental stages of *D. glomerata*

Vernalization is a process of phase transitions from vegetative growth to reproductive growth under ambient stimulation, specifically by low temperatures [29] and is programed and controlled by a large number of dynamically expressed genes including miRNA. Three up-regulated miRNAs (miR395, miR530, and miR167) and two down-regulated miRNAs (miR396 and miR528) in the VE-BV stage were most likely the main players in controlling vernalization. The expression of miR395 was negatively correlated with temperature and altered significantly in response to chilling stress. A previous study has demonstrated that miR395 was involved in BR signal response [30]. One target of miR395, *ABA*-receptor (*ABAR*), is a crucial element in the ABA signaling pathway [31]. Our results showed that the expression of miR395 and two BR signaling components, *BRI1* and *BZR1*, were up-regulated while *ABAR* was down-regulated from the BV to VE stage. This supports that miR395 is probably involved in vernalization through regulating BR and ABA signaling. Compared with miR395, the expression of miR530 was up-regulated in both the VE-BV and HT-BH comparisons. Nine miR530 targets were identified, including an indole biosynthetic process related gene (Enolase 1, *ENO1*) (Additional file 3). Loss of function of *ENO1* exhibited retarded vegetative growth, disturbed flower development, and seed abortion [32]. Thus, flowering time may be influenced by the inhibition of ENO1 through highly expressed miR530 in the VE stage.

MiR160 and miR167 are two critical miRNAs via crosstalk with the hormone auxin in plants [33]. MiR167 is thought be involved in floral development and the transition of vegetative and generative development [34], and miR160 plays a role in maintaining the process of normal developmental programs [35, 36]. MiR160 and miR167 converge on *ARFs*, which are involved in auxin and defense responses and are involved in many developmental stages. Specifically, miR167 mediates floral development by targeting *ARF6* and *ARF8* [37, 38], while miR160 negatively regulates *ARF10*, *ARF16*, *ARF17*, and *ARF18* in *Arabidopsis* [12, 39–41]. During the transition from the BV stage to the VE stage, miR160 and miR167 were oppositely expressed, and both of these miRNAs has varied expression in the VE stage. Even more noteworthy, the expression level of miR160 was positively correlated with temperature change, while miR167 was inversely correlated with temperature change. Out of 24 *ARFs* identified in our transcriptome data, the pattern of expression level changes of *ARF11*, *ARF25*, *ARF1*, *ARF12*, *ARF5*, *ARF7*, and *ARF17* were similar to the expression level changes of *ARF6*, indicating their possible functions in controlling flowering time. Therefore, we assumed that temperature regulated *ARFs* through miR160 and miR167, thereby affecting flowering time.

MiR396 promotes cell proliferation by targeting the *GROWTH-REGULATING FACTOR (GRFs)* in various plant organs [42, 43]. Meanwhile, miR396 was a positive regulator in response to drought stress in tobacco [44]. Recent research revealed that when *GRF4* relieved suppression by osa­miR396, the expression of *GRF4* accumulates to a proper extent and increases grain size by activating specific BR responses [45]. The suppression of *GRF* by miR396 was no longer present after vernalization, which probably accelerates cell division and differentiation with the increase in temperature during the transition from the VE stage to the AV stage. A study conducted by Yuan et al., [46] suggested that the potential crosstalk of miR528, miR172, miR156, and miR396 could contribute to orchestrating response to stress, plant phase transition, and flowering. It is possible that both the up-regulation of miR395, miR530, and miR167 and the down-regulation of miR396 and miR528 are mediated by temperature and associated with vernalization, determining the transition under adverse conditions in *D. glomerata*.

MiR156 and miR172 are the two key regulators inducing juvenile-to-adult transition that have antagonistic regulation [47]. MiR156 is required for the expression of juvenile leaf characteristics and repressing the expression of *SPLs* to prevent the change from the vegetative phase [10]. The MiR156-SPL3 module regulates *FT* to control ambient temperature-responsive flowering [48]. Recently, Hyun et al., [49] has demonstrated that *SPL15* cooperated with *SOC1* to coordinate floral initiation by activating the transcription of target genes. In turn, miR172 is activated by *SPLs* to promote the adult phase via the repression of *APETALA2*-like genes such as *AP2*, *TOE1*, *TOE2*, *SNZ*, and *SMZ* [50, 51]. In *D. glomerata*, the expression of miR156 was higher than that of miR172 at the BV and VE stages, while it was suppressed by miR172 at the AV, BH, and HT stages. The expression of miR156 gradually decreased during the transition from the BV stage to the HT stage, which is inversely correlated with the expression of miR172. In our data, five *AP2*-like unigenes were identified as floral homeotic protein *APETALA2* with a high expression level at the VE stage. The expression levels of nine SPL transcription factors (*SPL2*, *SPL3*, *SPL7*, *SPL8*, *SPL9*, *SPL13*, *SPL15*, *SPL16*, and *SPL17*) showed inverse correlation with the expression level of miR156, except for *SPL7* and *SPL9*. MiR166 and miR168 are also related to growth and developmental phase shift as identified in this study and reported previously [52, 53]. These observations indicated that four miRNAs, miR156, miR172, miR160, and miR168, may have a critical function in the transition from vegetative to reproductive phase after vernalization.

Three differentially expressed miRNAs, miR162, miR397, and miR398, related to stress response were observed in the comparison of the AV-VE stages. MiR162 is known to be involved in drought and salinity response in *Arabidopsis* [54]. Meanwhile, miR397 and miR398 were involved in the induction of freezing tolerance. Over-expression of miR397 improved plant tolerance to chilling stress by affecting the expression of *CBF* genes and *COR* genes [55]. In rice, the overexpression of osa­miR397 increases grain size by down-regulating LAC, which negatively regulates the sensitivity to BR signaling [56]. The expression of miR398 was regulated by the inducer of CBF expression (*ICE*) family genes, and its target genes copper/zinc superoxide dismutase 1 (*CSD1*) and *CSD2* inducible expression under acclimation at 16 °C [57]. Subsequent studies have shown that miR398 was heat-inhibitive and negatively regulated the expression of *HSFs* and *HSPs* [58]. In some plants, both chilling and heating was necessary for flowering [59]. Recent research has demonstrated that *HSP90* plays an extensive and essential role during the transition from the vegetative phase to the reproductive phase and flower development [60]. In our data, 54 *HSFs* and *HSPs* were detected in HT-BH. The expression profile of these DEGs was consistent with temperature change and the highest expression levels were found during the heading stage. This result indicated that the *HSF* and *HSP* gene families may participate in flowering in *D. golmerata*.

### Gene coexpression during vernalization

**Fig. 7:**
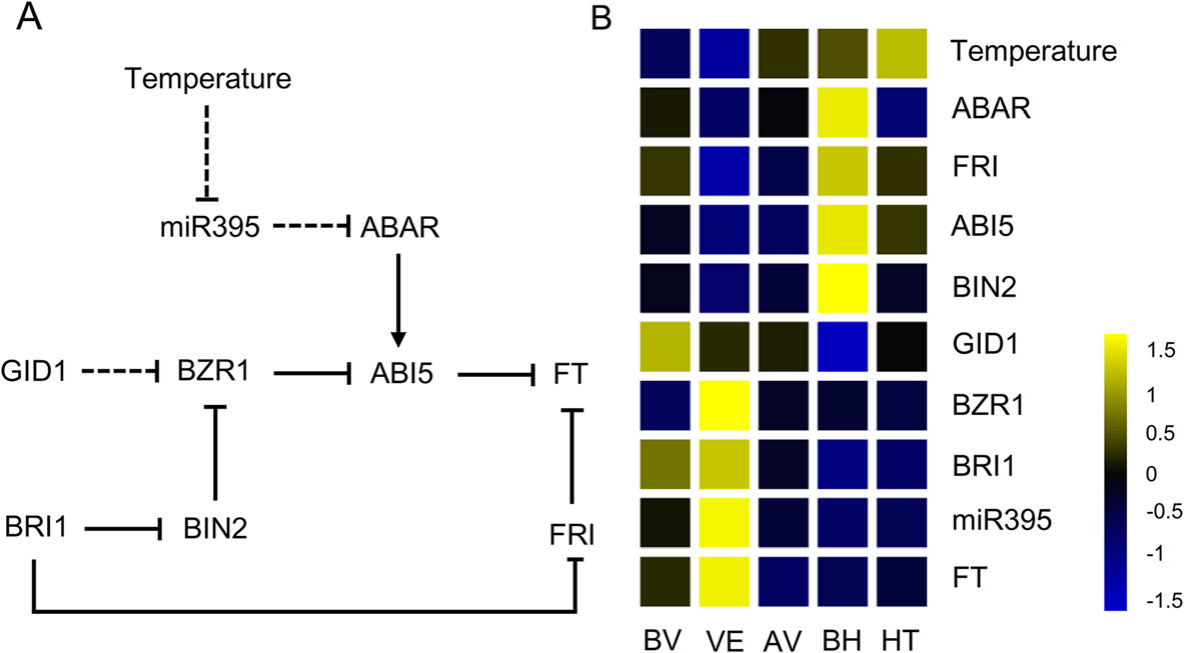
Analysis of gene expression in plant hormones pathways from growth stages BV to HT. **a** The putative network between GA, ABA and BR pathway. Line with arrows indicate positive regulation and line with blunt end indicated negative regulation. Dashed indicated the potential regulation. **b** The heatmap showed the gene expression involved in the network

Five hub genes, *BRI1*, *BZR1*, *VRN1*, *VIN3*, and *FT*, might play significant roles in regulation of vernalization response. Genes related to transporting, stress response, and plant hormones were detected in a subnetwork, which may have direct or indirect connections. Previous research has indicated that flowering time can be affected by BR signaling [61]. *BRI1* and *BZR1* are two vital nodes in the BR signaling pathway. *BRI1* mediated BR signaling leads to dephosphorylation and accumulation of *BZR1* in the nucleus [62]. In *Arabidopsis, BRI1* decreased the *FLC* expression by suppressing *FRI* to elevate the levels of the *FT* transcripts, thus accelerating floral transition [63]. Similarly, our investigation indicated that the expression of *BRI1* and *FT* were up-regulated while *FRI* was inhibited in vernalization response (Fig. 7). *BRI1* and *BZR1* were potentially relevant to vernalization in *D. golmerata*. BR-regulated processes largely interact with other plant hormone signaling [64]. For instance, *ABSCISIC ACID-INSENSITIVE 5* (*ABI5*) is dual modulated by *BIN2* and *BZR1* in BR signaling [65]. *BIN2* phosphorylates and stabilizes *ABI5* while *BZR1* suppresses the expression of *ABI5*, which makes plants less sensitive to ABA [66]. Wang et al. reported that the direct binding of *ABI5* to the promoter of *FLC* is a critical step for promoting *FLC* transcription, which inhibits *FT* expression and causes the postponement of flowering time [67]. *ABI5*, as the mutual downstream target, suggested a possible cross-regulatory relation between BR and ABA signaling in regulating flowering [68]. Gibberellins (GAs) play an important role in regulating diverse developmental processes in plants such as vegetative growth, floral induction, and flower development [69]. In GA signaling, *GIBBERELLIN INSENSITIVE DWARF1* (*GID1*) and *DELLAs* constitute negative feedback due to the influence of *DELLAs* on the expression of several genes [70, 71]. The BR and GA signaling processes converge at the *BZR1* and *DELLA* interaction by the formation of a BZR1-DELLA complex, which reduces the transcriptional ability of *BZR1* [71]. In our data, DELLA protein *SLN1* and *GAI* coexpressed with *BZR1* in the VE stage, providing evidence for BR-GA cross-talk. Hormone signaling has been shown to be interdependent and synergistic in many plant processes [64], and our research further illustrated the extensive cross-talk between different hormones in vernalization response.

Timely flowering initiation was control by the complex endogenous triggers and external environment. In temperate cereals, *VRN1* was homologous to the *Arabidopsis* meristem identity genes APETALA1 and VIN3-like, which were also identified in diploid wheat (*Triticum monococcum* L.) [4, 72]. In this study, three critical flowering-related genes *VRN1*, *VIN3*, and *FT* were identified in *D. glomerata* with up-regulated pattern when exposed to low temperature, suggests a certain degree of evolutionary conservation of flowering network between annuals and perennials. However, past research has revealed a differentiation in vernalization response between *A. thaliana* and cereal crops [3]. The vernalization response in temperate grasses is conferred by high expression levels of *VRN2* [7], which represses the *FT* ortholog *VRN3* [73, 74]. A more critical difference in the vernalization pathways is the absence of clear homologues of VRN2 in *A. thaliana* [75]. In addition, homologue of *FLC* in temperate cereals also not been identified. Based on genome synteny studies, several putative *FLC*-like genes have been detected in monocots [76], but less functional verification of *FLC* orthologs reported in cereals thus far [77]. This study may supply useful information for identifying genes related to vernalization response in temperate grasses.

## Conclusions

Our study displayed a dynamic variation of transcriptional and post-transcriptional regulation in vernalization and the heading stage of perennial grass. A total of 3,846 significant DEGs and 69 differentially expressed miRNAs were identified among five stages. The target genes for vernalization-responsive miRNAs functioned in transmembrane transport, signal transduction, and plant morphogenesis. One hundred and eighteen coexpressed genes formed a subnetwork, of which five hub targets, *BRI1*, *BZR1*, *VRN1*, *VIN3*, and *FT*, might provide new insights in the control of flowering time. These results promote a comprehensive understanding and elucidation of miRNA-mediated molecular mechanisms during vernalization, which may facilitate the molecular breeding in cereal crops and forages.

## Methods

### Plant materials

The seeds of orchardgrass variety BAOXING (Registered No. 197) were obtained from the Department of Grassland Science, Sichuan Agricultural University. The plants were grown in pots under natural environmental conditions in Sichuan Agricultural University, Chengdu, Sichuan Province, on 21 September 2016. Mixed samples of young leaves were collected and immediately frozen in liquid nitrogen. Five sampling points were chosen: before vernalization (BV, 4 January 2016), vernalization (VE, 2 February 2016), after vernalization (AV, 2 March 2016), before heading (BH, 24 March 2016), and heading (HT, 9 April 2016) (Additional file 5: Figure S3). At each sampling point, three biological replicates were collected. Total RNA was extracted from the mixed samples using the RNAprep Pure Plant Kit (Tiangen Biochemical Technology Company, Beijing, China). RNA purity and concentration was measured via the NanoPhotometer® spectrophotometer (IMPLEN, CA, USA) and Qubit® RNA Assay Kit in Qubit® 2.0 Flurometer (Life Technologies, CA, USA). RNA integrity was assessed using the RNA Nano 6000 Assay Kit and Agilent Bioanalyzer 2100 system (Agilent Technologies, CA, USA). Floral primordium was collected to investigate the inflorescence formation after the induction of vernalization. The floral primordium was excised and immediately fixed in Carnoy’s Fluid. After fixation, tissues were routinely dehydrated and embedded with paraffin. Thin sections (5 μm) of each tissue were sliced from each block and mounted on glass. Then, the sections were stained with hematoxylin. Images were recorded using a Leica DM4 B-microscope system (Leica, Germany).

### Transcriptome sequencing and de novo assembly analysis

Approximately 3 μg RNA per sample was used for transcriptome library preparation, and a total of 15 libraries were generated using NEBNext® Ultra™ Directional RNA Library Prep Kit for Illumina® (NEB, USA) following the manufacturer’s protocols. First strand cDNA was synthesized using random hexamer primer and M-MuLV Reverse Transcriptase (RNase H-), then second strand cDNA was synthesized using DNA polymerase I and RNase H. The AMPure XP system (Beckman Coulter, Beverly, USA) was used to select cDNA fragments preferentially 150–200 bp in length. Products were purified using the AMPure XP system and library quality was assessed on Agilent Bioanalyzer 2100 system. Transcriptome sequencing was performed on an Illumina HiSeqTM 2000 platform. Trinity (http://trinityrnaseq.github.io/) was used to assemble high-quality reads into nonredundant unigenes. All the assembled unigenes were searched and annotated against the publicly available protein databases including Nr, Nt, Pfam, KOG/COG(EuKaryotic Orthologous Groups/ Clusters of Orthologous Groups, Swiss-prot, KEGG(Kyoto Encyclopedia of Genes and Genomes), and GO (Gene Ontology), using BLASTx analysis with an E-value cut-off of 1.0E-05. Genes were thus tentatively identified according to the best hits against known sequences. The expected number of fragments per kilobase of transcript sequence per million base pairs sequenced (FPKM) was used to estimate the gene expression levels. Differentially expressed genes (DEGs) of five pairwise sample comparisons including VE vs BV, AV vs VE, BH vs AV and HT vs BH were identified applying the cutoff |log2FC| > 1, *P* < 0.01 using DESeq Rpackage (1.18.0) software [78, 79].

### Small RNA sequencing and identification of microRNAs

To generate small RNA libraries, approximately 3 μg of RNA (RIN number > 7.0) per sample from the five sampling points was used. Sequencing libraries were generated using NEBNext® Multiplex Small RNA Library Prep Set for Illumina® (NEB, USA) following the manufacturer’s protocols. After PCR amplification, the products were purified on an 8% polyacrylamide gel (100 V, 80 min). Fragments corresponding to 140–160 bp were recovered and dissolved in 8 μL elution buffer. Library quality was assessed on the Agilent Bioanalyzer 2100 system using DNA High Sensitivity Chips. Sequencing was performed on Illumina Hiseq 2500/2000 platform for raw reads in 15 sRNA libraries. Clean reads were obtained by removing the contaminated sequences (5′ adapters), reads without 3′ adapters and insert, low-quality sequences (the reads with sQ ≤ 5 base more than 50%), reads containing poly-N(> 10%), and poly-A/T/G/C. Reads smaller than 18 nt also removed through Illumina’s Genome Analyzer Pipeline V1.5. After trim the 3′ adapters, The remaining clean reads were mapped to *D. glomerata* reference sequence to analyze their expression and distribution by Bowtie [80]. MiRBase20.0 (http://www.mirbase.org/) was used to look for known miRNA. MiREvo and mirdeep2 were integrated to predict novel miRNA [81, 82]. The identified, known miRNAs were searched against miFam.dat (http://www.mirbase.org/ftp.shtml) to identify their families. Novel miRNA precursors were submitted to Rfam (http://xfam.org/) to look for Rfam families. Target genes of miRNA were predicted using psRobot_tar in psRobot [83]. MiRNA expression levels were estimated by TPM (transcript per million) using the following formula: Normalized expression = mapped read count/ (total reads*1,000,000) [84]. Differential expression analysis was performed using DESeq in R (1.8.3) [78]. The *P*-values were adjusted according to the Benjamini and Hochberg method. The significantly differentially expressed genes were identified based on the following thresholds: *P*-value ≤0.01, false discovery rate ≤ 0.01, and |log2 ratio| ≥ 1. Goseq and KOBAS software were implemented for enrichment analyses (http://www.genome.jp/kegg/) [85, 86]. Weighted gene coexpression network analysis was performed by WGCNA package in R (v3.3.0) as previously described [87]. Transcripts with FPKM values above 0 in more than two duplications were chosen for the WGCNA analysis [88, 89]. Gene expression adjacency matrix was constructed to analyze the network topology with the soft threshold power set to 10, TOMType as unsigned, minModuleSize as 30, and mergeCutHeight as 0.25 in the analysis. The blockwiseModules were used to obtain the modules using the default setting. The networks were visualized using Cytoscape v.3.0.0. The heat map was obtained using OmicShare tools (www.omicshare.com/tools). All the sequencing data was deposited into the NCBI SRA database under the accession numbers SRP131840 and SRP131899.

### Verification by qRT-PCR

The validity of transcriptome and miRNA sequences was verified by quantitative real-time PCR (qRT-PCR). Eight flowering related genes (*LHY* (c146679_g3), *FRI* (c132929_g2), *LHP1* (c143664_g4), *FD* (c128431_g1), *VRN1* (c147469_g1), *SPL9* (c150483_g1), *GI* (c151406_g1), and *SPL3* (c134262_g2)) and eight differentially expressed miRNAs (miR528, miR408, miR399, miR398, miR397, miR166, miR167, and miR172) were selected for validation. Primers were designed from the candidate gene sequences using the online Primer BLAST program (https://www.ncbi.nlm.nih.gov/tools/primer-blast) (Additional file 3). Glyceraldehyde 3-phosphate dehydrogenase (GAPDH) and U6 were selected as reference genes [90, 91]. The detailed methods have been described in our previous study [92]. First-strand cDNAs of miRNAs were synthesized using Mir-X™ miRNA FirstStrand Synthesis Kit (Code No. 638315) (Takara, Japan), and the qRT-PCR reaction was performed using Bio-Rad CFX96 following the instructions for the SYBR® Premix Ex Taq™ II (Tli RNaseH Plus) (Code No.RR820Q) (Takara, Japan). Three replicates were performed on each sample. Relative quantitative level were calculated based on 2^-ΔΔCT^ method [93].

## Additional files

Additional file 1: **Table S1.** Summary statistics of RNA sequencing in different samples. **Table S2.** Summary of transcriptome sequencing for *D. glomerata*. **Table S3.** Summary statistics of the quality of sRNA sequencing. **Table S4.** Summary statistics of sRNA sequencing in samples from different stages. **Table S5.** Summary statistics of sRNA mapping results. **Table S6.** Length distribution of total sRNA. **Table S7.** Summary statistics and Length distribution of known miRNAs and novel miRNAs. **Table S8.** Statistics of differentially expressed miRNAs. **Table S9.** The targets of differentially expressed miRNAs. **Table S10.** Statistics for significant DEGs. (XLSX 313 kb)

Additional file 2: **Figure S1.** Characterization of identified miRNAs from *D. glomerata*. (A) The first nucleotide of known miRNAs. (B) The first nucleotide of novel miRNAs. (TIF 1545 kb)

Additional file 3: **Table S11**. The primers used for qRT-PCR. **Table S12**. Targets of miRNAs in the BV stage, VE stage, and AV stage. **Table S13**. Statistics for GO enrichment results. **Table S14**. DEGs in the VE-BV-UP vs AV-VE-DOWN and VE-BV-DOWN vs AV-VE-UP comparisons. **Table S15**. Concerned DEGs in the VE-BV, AV-VE, and HT-BH comparison. **Table S16**. The genes in the brown-module. **Table S17**. The targets of miR530. (XLSX 36 kb)

Additional file 4: **Figure S2.** WGCNA of DEGs in different stages. (A) Hierarchical cluster tree showing coexpression modules identified by WGCNA. Each leaf in the tree is one gene. The major tree branches constitute 12 modules labeled by different colors. (B) Module-stage association. Each row corresponds to a module. Each column corresponds to a specific stage. The color of each cell at the row-column intersection indicates the correlation coefficient between the module and the stage. A high degree of correlation between a specific module and the stage is indicated by dark red. (TIF 10484 kb)

Additional file 5: **Figure S3.** The definition of sampling point and the photographs of *D.glomerata* at five developmental stages. (TIF 11006 kb)

## Abbreviations

ABA: Abscisic acid
ABI5: ABSCISIC ACID-INSENSITIVE 5
ARF: Auxin response factor
AV: After vernalization
BH: Before heading
bHLH: Basic helix-loop-helix
BR: Brassinosteroid
BRI1: BRASSINOSTEROID INSENSITIVE1
BV: Before vernalization
BZR1: Brassinazole BRASSINAZOLE RESISTANT 1
CIR1: Circadian 1
CO: CONSTANS
COG: Cluster of orthologous group
DEGs: Differentially expressed genes
ERF: Ethylene-responsive transcription factor
FLC: Flowering locus C
FPKM: Fragments Per Kilobase of transcript sequence per Million base pairs sequenced
FRI: FRIGIDA
FT: Flowering locus T
GA: Gibberellin
GO: Gene Ontology
HSP: Heat shock protein
HT: Heading stage
IAA: Indole-3-acetic acid
ICE1: Inducer of CBF expression 1
KEGG: Kyoto Encyclopedia of Genes and Genomes
KOBAS: Kegg Orthology Based Annotation System
KOG: Eukaryotic orthologous groups
LAF1: Long after far-red light 1
MADS: MCM1, AGAMOUS, DEFICIENS and SRF
MYB: Myeloblastosis, a transcription factor family
NAC: NAM, ATAF1,2, CUC2
Nr: Non-redundant protein sequence
Pfam: Protein family
qRT-PCR: Quantitative real-time PCR
SOC1: Suppressor of overexpression of CO1
SPL3: SBP-box gene family squamosa promoter binding protein-like3
Swiss-prot: Annotated protein sequence database
TFs: Transcription factors
TPM: Transcript per million
VE: vernalization
VIN3: Vernalization insensitive 3
VRN: Vernalization
WGCNA: Weighted correlation network analysis
