# Supplementary figures and images for: Integration of small RNAs and transcriptome sequencing uncovers a complex regulatory network during vernalization and heading stages of orchardgrass (Dactylis glomerata L.)
Source: BMC Genomics. 2018 Oct 3;19:727. doi: 10.1186/s12864-018-5104-0 (PMC6171228; doi:10.1186/s12864-018-5104-0)

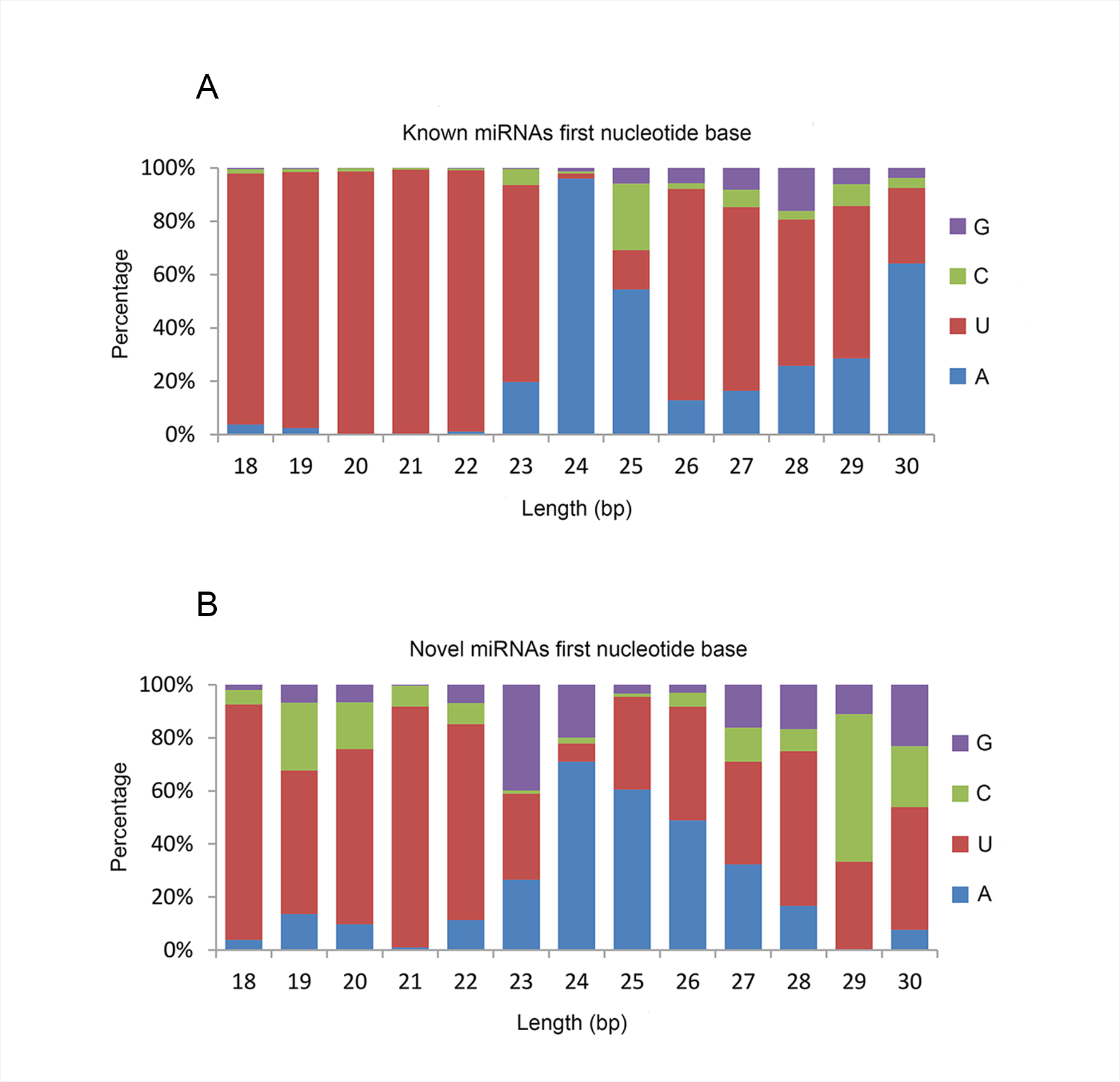

Supplement: Supplementary file 2 — Figure S1. Characterization of identified miRNAs from D. glomerata. (A) The first nucleotide of known miRNAs. (B) The first nucleotide of novel miRNAs. (TIF 1545 kb) [file 12864_2018_5104_MOESM2_ESM.tif]

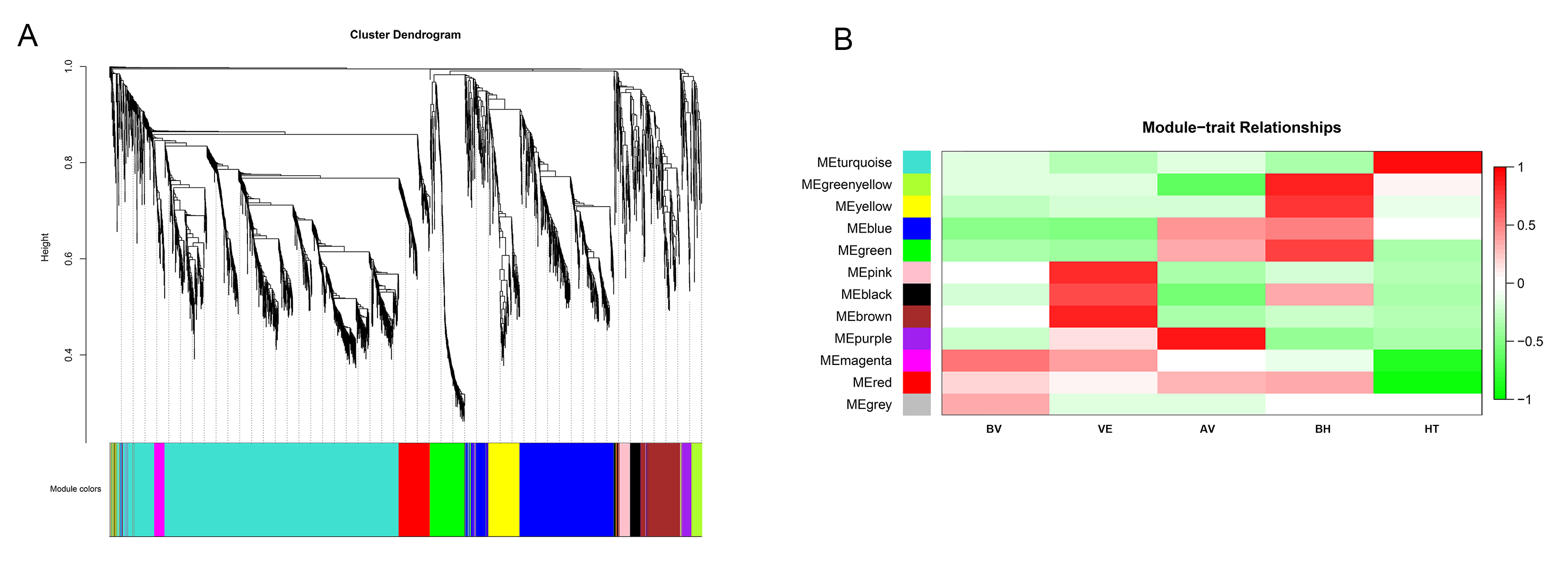

Supplement: Supplementary file 4 — Figure S2. WGCNA of DEGs in different stages. (A) Hierarchical cluster tree showing coexpression modules identified by WGCNA. Each leaf in the tree is one gene. The major tree branches constitute 12 modules labeled by different colors. (B) Module-stage association. Each row corresponds to a module. Each column corresponds to a specific stage. The color of each cell at the row-column intersection indicates the correlation coefficient between the module and the stage. A high degree of correlation between a specific module and the stage is indicated by dark red. (TIF 10484 kb) [file 12864_2018_5104_MOESM4_ESM.tif]

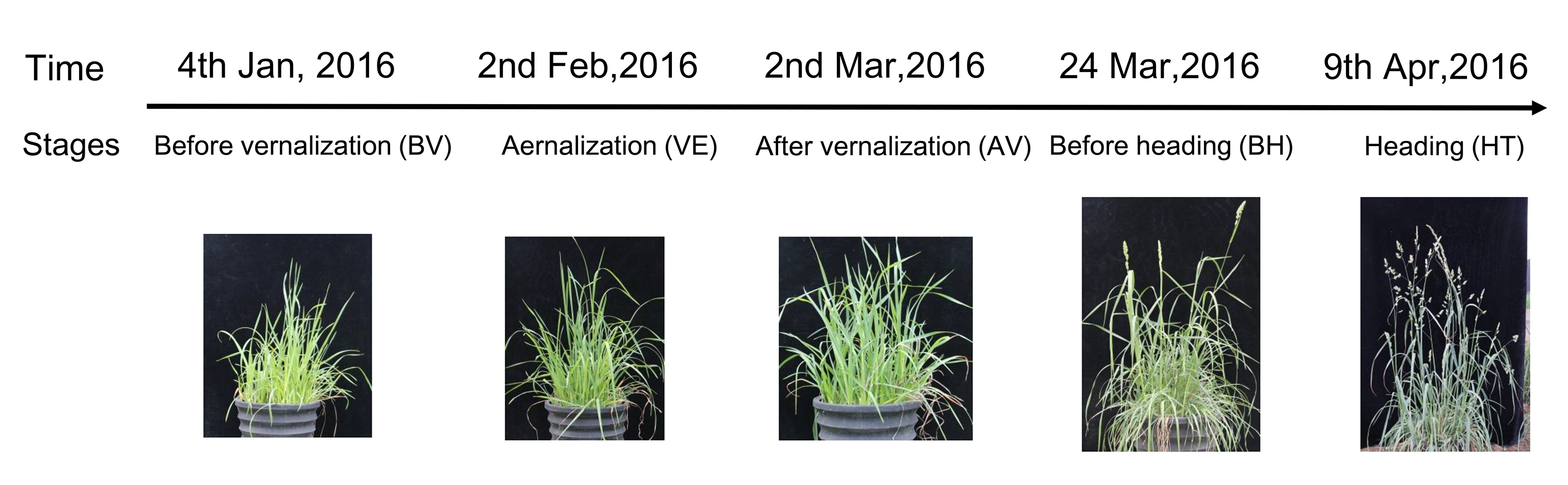

Supplement: Supplementary file 5 — Figure S3. The definition of sampling point and the photographs of D.glomerata at five developmental stages. (TIF 11006 kb) [file 12864_2018_5104_MOESM5_ESM.tif]
